# Constructing comparable intimate partner violence indicators across the DHS, MICS, and PMA health surveys

**DOI:** 10.1057/s41271-024-00503-3

**Published:** 2024-07-12

**Authors:** Devon Kristiansen, Maya Luetke, Matt Gunther, Miriam King, Anna Bolgrien, Mehr Munir

**Affiliations:** 1 Institute of Social Research and Data Innovation, University of Minnesota-Twin Cities, Minneapolis, MN USA; 2 NORC, University of Chicago, Chicago, IL USA

**Keywords:** Comparability, Harmonization, Intimate partner violence

## Abstract

We construct comparable indicators that measure the prevalence of recent intimate partner violence (IPV) using publicly available, integrated microdata within the IPUMS data collections across many countries. The objective of this work is to increase opportunities for comparative research by leveraging vast quantities of harmonized data. We use consistent and comparable variables that measure domestic violence in international health surveys. The most consistent indicators of domestic violence measure physical, psychological, and sexual IPV in the last 12 months. We imposed a consistent reference period and restricted to a comparable subpopulation where these differed across surveys. Aggregating IPV variables across surveys, without careful attention to question wording and subpopulations, may produce inconsistent measures leading to bias, over- or under-estimation of IPV prevalence, or spurious trends and associations. Using comparable indicators in microdata and studying the level, distribution, and covariates of IPV in multiple settings over time, we can better understand these phenomena and identify effective policy interventions.

## Key Messages

- We harmonized health data from several instruments - Demographic and Health Surveys (DHS), Multiple Indicator Cluster Surveys (MICS), and Performance Monitoring for Action (PMA) - to create intimate partner violence indicators.
- The constructed comparable intimate partner violence indicators offer both a substantive opportunity for intertemporal and cross-national comparative analysis and a framework for approaching the harmonization of other health indicators.
- Non-comparable indicators could lead to bias, over- or under-estimation of IPV prevalence, and/or spurious trends and associations.

## Objectives

We describe our approach to constructing comparable indicators that measure the prevalence of recent intimate partner violence (IPV) using publicly available, integrated microdata within the IPUMS data collections around the world. Our objective is to facilitate rigorous IPV research that can compare trends and relationships across geographical regions and over time.

IPUMS is an organization that disseminates harmonized and well-documented census and survey data. IPUMS Global Health is the world’s largest repository of free, harmonized global health survey data, which contains three components: the Demographic and Health Surveys (DHS), the Multiple Indicator Cluster Surveys (MICS), and Performance Monitoring for Action (PMA), currently representing 110 countries. Each component contains thousands of consistently coded and named variables about individuals (women, children), and other units of analysis, such as health facilities or households. The integrated microdata are downloadable in free custom datafiles from the IPUMS websites [1–3].

DHS, MICS, and PMA datasets contain hundreds of variables about domestic violence. The surveys follow standard protocols to ensure privacy and confidentiality when asking about respondents’ experiences, utilize similar eligibility criteria, and measure IPV consistently with global standards [4–6]. They differ in the broad topics and specific questions asked about domestic violence, which complicates cross-survey analyses. All three surveys asked about multiple manifestations of IPV, defined as abuse perpetrated by a woman’s husband or cohabiting partner. Other types of domestic violence are not our focus here, because they were not included in all the surveys.

## Methods

By identifying and combining variables with similar question wording across DHS, MICS, and PMA, we created three comparable compound variables that measure physical, psychological, and sexual IPV in the last 12 months.

To achieve comparability, we adjusted the subpopulation of women included. In PMA, only currently married or cohabiting women were asked IPV questions, whereas DHS and MICS asked ever-partnered women. DHS and MICS asked about violence ever experienced, with follow-up questions about the frequency in the past 12 months, while PMA only asked whether the behavior occurred in the past 12 months by the current partner. With a bit of coding, we easily imposed a consistent subpopulation and time frame. To assist other researchers, we have made our code available on the IPUMS Global Health GitHub page [7].

Another comparability issue arises when surveys offer summary measures that group abusive behaviors in different ways. PMA opted for a short set of physical IPV questions, combining multiple behaviors into a single query, while DHS and MICS asked separate questions about various violent behaviors. We created a new summary measure for physical IPV, whose components matched across the surveys.

For psychological IPV, DHS and MICS asked, among other questions, whether the partner “threaten[ed] her or someone close to her with harm”, while PMA did not. We therefore excluded this threatening behavior when grouping responses to create a comparable compound measure of psychological IPV.

The sexual IPV variables posed the most significant challenges to comparability. PMA, DHS, and MICS surveys differ in whether they asked about sexual acts obtained through physical force versus coercion without force. We could make only one comparable sexual abuse variable: physical forcing of sex in the past 12 months.

## Takeaways

**Fig. 1:**
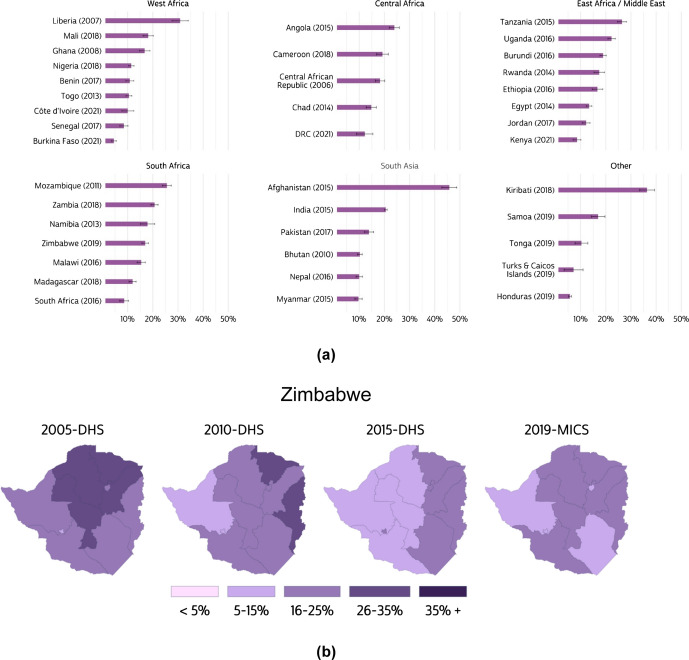
**a** Prevalence of physical intimate partner violence experienced in the past 12 months by currently partnered women, most recent sample by country (2006–2022); **b** Prevalence of physical intimate partner violence experienced in the past 12 months by currently partnered women in Zimbabwe, 2005–2019

Combining data from IPUMS DHS, MICS, and PMA surveys greatly expands the geographic coverage of analyses of IPV. Figure 1a maps the percent of currently partnered women age 15–49 who experienced physical IPV in the last 12 months, using our comparable indicator and the most recent data for each country.

Using microdata across multiple samples from different surveys allows researchers to observe temporal trends in the prevalence of IPV disaggregated to subnational levels. Using the same measure, Fig. 1b shows a downward trend in the prevalence of physical IPV by region in Zimbabwe, according to DHS and MICS data. Because subnational administrative boundaries sometimes change over time, geographers at IPUMS have created variables that represent comparable geographic areas, combining regions where necessary to create consistent boundaries over time. We provide corresponding shapefiles on our website.

In sensitivity analyses, we found that results from using our comparable indicators described above differ significantly from results from more inclusive indicators, constructed by aggregating all the behaviors for each type of IPV (physical, psychological, and sexual) covered by the three surveys. Estimates of national psychological IPV prevalence using a non-comparable indicator that sums all such behaviors, regardless of comparability across survey types, can differ significantly from the comparable indicator we constructed. For example, the national estimate for Jordan 2007 (DHS) for the non-comparable indicator is 14%, but the estimate is 5.5% using the comparable indicator.

## Conclusions

Aggregating IPV variables across surveys without careful attention to question wording and universes may produce non-comparable measures leading to bias, over- or under-estimation of IPV prevalence, or spurious trends and associations. Researchers should carefully consider the inputs for survey-created summary variables when combining IPV data across surveys. While dashboards and summary statistics have their uses, microdata on IPV (as well as other topics) offer unique possibilities for analysis. Using microdata, such as that freely available from IPUMS Global Health, one can look for trends or associations using multivariate analysis, or focus on specific subsamples, such as rural or adolescent populations. By studying the level, distribution, and covariates of IPV in multiple settings over time, we can better understand these phenomena and identify effective policy interventions.

## Data Availability

IPUMS DHS—Integrated Demographic and Health Surveys data, currently covering Africa and South Asian surveys from the 1980s to the present. Custom data downloads and systematic documentation are available for free to authorized users. IPUMS MICS—Integrated Multiple Indicator Cluster Survey data covering health and well-being for 88 countries. Custom data downloads and systematic documentation are available for free to authorized users. IPUMS PMA—Integrated Performance Monitoring for Action data on fertility, contraception, hygiene, and health for 11 countries in Asia and Sub-Saharan Africa. Custom data downloads and systematic documentation are available for free to authorized users. IPUMS Global Health GitHub, Interoperability-domestic-violence-data repository—Stata code available to create the comparable IPV indicators described in this brief using custom data extracts downloaded from IPUMS DHS, IPUMS MICS, or IPUMS PMA websites.
